# Thymoquinone-Loaded Soluplus^®^-Solutol^®^ HS15 Mixed Micelles: Preparation, In Vitro Characterization, and Effect on the SH-SY5Y Cell Migration

**DOI:** 10.3390/molecules25204707

**Published:** 2020-10-14

**Authors:** Maria Camilla Bergonzi, Marzia Vasarri, Giulia Marroncini, Emanuela Barletta, Donatella Degl’Innocenti

**Affiliations:** 1 Department of Chemistry, University of Florence, Via Ugo Schiff 6, Sesto Fiorentino, 50019 Florence, Italy; giulia.marroncini1@stud.unifi.it; 2 Department of Experimental and Clinical Biomedical Sciences “Mario Serio”, Viale Morgagni 50, 50134 Florence, Italy; marzia.vasarri@unifi.it (M.V.); emanuela.barletta@unifi.it (E.B.); donatella.deglinnocenti@unifi.it (D.D.)

**Keywords:** thymoquinone, polymeric micelles, Soluplus^®^, Solutol^®^ HS15, solubility, stability, neuroblastoma, cell migration, wound-healing assay

## Abstract

Thymoquinone (TQ) is the main active ingredient of *Nigella sativa* essential oil, with remarkable anti-neoplastic activities with anti-invasive and anti-migratory abilities on a variety of cancer cell lines. However, its poor water solubility, high instability in aqueous solution and pharmacokinetic drawbacks limits its use in therapy. Soluplus^®^ and Solutol^®^ HS15 were employed as amphiphilic polymers for developing polymeric micelles (SSM). Chemical and physical characterization studies of micelles are reported, in terms of size, homogeneity, zeta potential, critical micelle concentration (CMC), cloud point, encapsulation efficiency (EE%), load capacity (DL), in vitro release, and stability. This study reports for the first time the anti-migratory activity of TQ and TQ loaded in SSM (TQ-SSM) in the SH-SY5Y human neuroblastoma cell line. The inhibitory effect was assessed by the wound-healing assay and compared with that of the unformulated TQ. The optimal TQ-SSM were provided with small size (56.71 ± 1.41 nm) and spherical shape at ratio of 1:4 (Soluplus:Solutol HS15), thus increasing the solubility of about 10-fold in water. The entrapment efficiency and drug loading were 92.4 ± 1.6% and 4.68 ± 0.12, respectively, and the colloidal dispersion are stable during storage for a period of 40 days. The TQ-SSM were also lyophilized to obtain a more workable product and with increased stability. In vitro release study indicated a prolonged release of TQ. In conclusion, the formulation of TQ into SSM allows a bio-enhancement of TQ anti-migration activity, suggesting that TQ-SSM is a better candidate than unformulated TQ to inhibit human SH-SY5Y neuroblastoma cell migration.

## 1. Introduction

Neuroblastoma (NB) is by far the most common extracranial solid tumor diagnosed in infancy, with an average age of 1–2 years at the diagnosis. It is a neuroendocrine tumor that originates from the developing sympathetic nervous system and with a highly variable clinical picture, depending on the stage and location of the tumor. It accounts for 7% of all childhood cancers with nearly 500 new cases each year and 15% of all cancer deaths in the pediatric population [1]. Generally, the risk of many cancers in adults can be reduced with some lifestyle changes (such as an adequate dietary intake and exercise or stopping smoking and alcohol abuse), but currently, there is no way to prevent most cancers in children. At present, there are no causes of neuroblastomas linked to lifestyle or the environment, while the only known risk factors (age and heredity) cannot be changed. Moreover, despite the clinical progress with multimodal intensive care, including surgical resection, chemotherapy, immunotherapy, radiotherapy, myeloablative treatment, or retinoid therapy, these treatment options are often toxic and not always effective [2,3]. As a matter of fact, more than half of children with high-risk aggressive NB are still suffering from refractory or relapsing diseases developing widespread metastasis, especially in liver, non-contiguous lymph nodes, bone marrow, and other organs [4]. Mostly of patients with refractory or relapsed disease the lack of curative treatment solutions makes the overall mean survival extremely low [5]. Therefore, new innovative approaches are urgently needed to avoid refractory or relapsing disease and to increase the rate of survival. Among various therapeutic options, targeting tumor cell invasion and migration is a possible strategy against tumor progression [6].

Currently, there is a general tendency to select new anticancer agents from natural products because they are generally related to relatively less toxicity and minimal side effects. Over the years, the study of traditional or popular medicine along with modern medicine has allowed researchers to identify active compounds for the development of functional foods for strategic dietary interventions aimed at preventing the progression of chronic diseases, such as cancer metastases [7,8].

**Figure 1:**
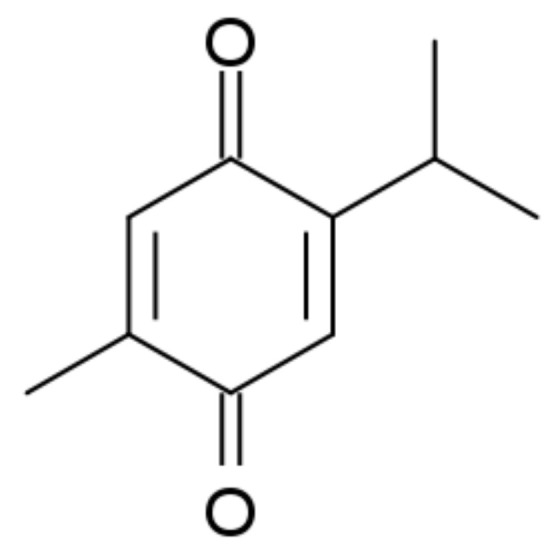
Structure of thymoquinone.

Rich in history and tradition, *Nigella sativa* L. (black cumin), belonging to the Ranunculaceae family, is a plant today known as “miraculous herb” for its wide range of pharmacological potential [9]. The folkloristic use of black cumin seeds and their oil as food or herbal drug preparation in medical practices against various human diseases was particularly widespread in South and Southeast Asia, Africa, Arabia, and in the Mediterranean regions. Most of the beneficial pro-health properties of this plant are attributed to thymoquinone (2-isopropyl-5-methyl-benzo-1,4-quinone, TQ) (Figure 1), the most important active ingredient of black cumin essential oil [9,10,11].

TQ has versatile bioactive properties, with antioxidant, anti-inflammatory, immunomodulatory, antihistaminic, analgesic, antidiabetic, antimicrobial, and antiviral effects. The compound also has beneficial effects in cardiovascular, reproductive, diabetes, respiratory, and neurological disorders, as well as in the management of bone complications and fibrosis. Moreover, TQ has shown remarkable anti-neoplastic activities with anti-invasive and anti-migratory abilities on a variety of cancer cells, including human glioblastoma and murine neuroblastoma cells [12,13], colon cancer cells [14], breast cancer cells [15], human non-small cell lung cancer cells [16], vascular smooth muscle cells [17] and cervical cancer cells [18]. Furthermore, many studies agree that TQ has no significant adverse effects and no serious toxicity [19,20]. Despite its great nutraceutical potential, TQ is poorly soluble in water (550 μg/mL), unstable in aqueous solutions, particularly at an alkaline pH, and it has severe light sensitivity [21]. TQ has shown high instability, rapid elimination and a great ability to bind to plasma membranes limiting the effectiveness of its beneficial effect [21] and, consequently, its clinical use [22]. Then, the aqueous solutions are inappropriate as pharmaceutical vehicles for TQ preparations. Therefore, the focus on nanotechnology to improve bioavailability and drug delivery is of growing importance. The use of new alternative therapeutic delivery systems, such as nanocarriers, can improve efficacy and reduce systemic toxicity during the treatment of malignant tumors compared to the use of “free” drugs [23].

To overcome the drawbacks of TQ, several strategies have been employed including polymeric nanoparticles, lipid carriers, micro and nanoemulsions, Self-Emulsifying Drug Delivery Systems, cyclodextrin complexes, and liposomes. These nanocarriers ameliorate the bioavailability and the anticancer and anti-inflammatory activities of TQ when compared with free substance. The encapsulation in nanoparticles improves the delivery and limits undesirable cytotoxicity [24,25,26,27].

Among the varieties of nanoformulations available to improve biopharmaceutics properties of drugs, polymeric micelles (PM) are of great interest for pharmacological application [28]. Food and Drug Administration (FDA) approved Genexol^®^ PM, a micellar formulation of paclitaxel for the treatment of breast, ovarian and lung cancer [29].

Polymeric micelles are core/shell structures with nanoscopic dimensions, consisting of amphiphilic blocks copolymers formed by a hydrophilic (e.g., polyethylene glycol (PEG), polyvinylpyrrolidone (PVP)) and a lipophilic portion (e.g., polyesters, polyanhydrides, and polyaminoacids). They are particularly well suited for drug delivery purposes due to their inherent and modifiable properties. The advantages include solubilization of poorly soluble molecules, sustained release and size advantages, and protection of encapsulated substances from degradation and metabolism [30]. Such nanoparticulate systems are made of synthetic or natural polymers which can be modified to modulate the drug release kinetics. PM result safe and usable for parenteral administration, permitting a rise within the dose of potent yet toxic and poorly water-soluble compounds. Polymeric micelles may circulate for extended periods in blood and gradually release drug. Based on their constituents, they can inhibit P-glycoprotein at drug-resistant tumors, gastrointestinal tract, and blood/brain barrier, perhaps providing some way to beat drug resistance in cancer and increase drug absorption from the gut and drug absorption into the brain [31]. Many of these properties are connected to the hydrophilic portion of the PM, in particular the PEG moiety. The low cost, the limited toxicity and the high degree of hydration that stabilizes in an aqueous medium the nanostructured systems explain the wide use of this polymer [32,33].

**Figure 2:**
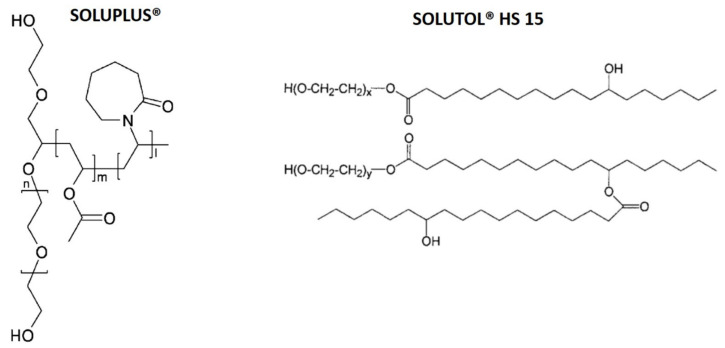
Chemical structures of Soluplus^®^ and Solutol^®^ HS15.

Among pegylated copolymers, Soluplus^®^ is a tri-block copolymer consisting of polyvinylcaprolactam-polyvinylacetate-polyethylene glycol (Figure 2). It has a low critical micelle concentration (CMC) and its micelles are highly stable after dilution [34,35]. It is an innovative excipient that gives new levels of solubility and bioavailability of poorly water-soluble drugs and natural compounds both for oral and parenteral administration [35,36,37,38,39,40,41,42,43,44,45]. Solutol^®^ HS15 (polyoxyethylene esters of 12-hydroxystearic acid, Figure 2) is a known non-ionic surfactant with high solubility and stability and low toxicity in vivo. It consists of a lipophilic and a hydrophilic portion, which comprise mono- and di-esters polyglycols of 12-hydroxystearic acid and about 30% of free polyethylene glycol, respectively. It is a multi-drug resistance modifying agent both in vitro and in vivo [46,47]. It alters the binding to plasma proteins, improving the adsorption behavior and inducing marked effects on the pharmacokinetics of numerous drugs [48]. Like Soluplus^®^, it is a solubilizer particularly suitable for parenteral and oral dosage forms [49,50,51,52].

Given the considerable variety of nanoformulations, described in the literature, available to improve biopharmaceutical properties of drugs, in this study for the first time the mixed Soluplus^®^–Solutol^®^ HS15 micelles (SSM) were applied to TQ delivery with the aim to increase its the aqueous solubility and bioactivity. The two polymers were also selected for their low toxicity and known use for parenteral administration.

Moreover, for the first time the anti-migratory capacity of TQ formulated in SSM (TQ-SSM) was investigated on the SH-SY5Y human neuroblastoma cell line. In a previous study, the authors have shown that the nanoformulation of *Posidonia oceanica* extract allows improvement of the extract inhibitory activity against cell migration [39], due to effect on the solubility of the extract and the modulation of its release.

Chemical and physical characterization studies, in terms of size, homogeneity, zeta potential, CMC, cloud point, encapsulation efficiency, load capacity, in vitro release, and storage stability, were performed on empty SSM and TQ-SSM. In addition, the stability of the formulations was investigated in vitro in blood conditions and the lyophilization was evaluated as a useful process to obtain a solid product. Finally, the inhibitory effect of TQ-SSM on SH-SY5Y cell migration was assessed by the wound-healing assay and compared with that of the unformulated TQ.

## 2. Results and Discussion

### 2.1. Preparation and Characterization of TQ-SSM

Polymeric micelles were developed to improve the aqueous solubility of TQ and to test the influence of the carrier on the inhibitory potential on cancer cell migration, providing a sustained and prolonged release. Different Soluplus^®^ and Solutol^®^ HS15 gravimetric ratios (1:1, 1:4, 2:1, 2:3, 4:3, 4:5) were considered to obtain a formulation with high encapsulation efficiency and with appropriate technological parameters for parenteral administration and stability features. Several amount of TQ, from 2 mg/mL to 6 mg/mL, were also considered. Nevertheless, tested formulations displayed adequate sizes between 50 and 60 nm with narrow distribution (PDI < 0.2) but many of them did not have good physical stability or high encapsulation efficiency.

Finally, the optimized formulation consisted of Soluplus:Solutol 1:4 gravimetric ratio (SSM) and it is able to load 5 mg/mL of TQ, with an EE% of 92.4 ± 1.6% and DL% of 4.68 ± 0.12. Moreover, the TQ presence did not affect the physical and chemical characteristics and the stability of empty micelles (Table 1). The TQ solubility is increased about 10-fold in water.

The freeze-drying process in the absence of cryoprotectant was also considered to increase the stability of the formulation (TQ-SSML) over a prolonged period and to obtain a more workable product. The freeze-dried process did not modify the size and homogeneity of the samples when the freeze-dried product was re-dispersed in water: the physical and chemical parameters before and after the process are substantially comparable (Table 1). The formulation is easily re-dispersible, without leaving undissolved solid. This is due to the presence of the two polymers that protect TQ and quickly rehydrate.

### 2.2. Self-Aggregation of Polymeric Micelles and Cloud Point

The CMC is an essential parameter influencing the stability of the micelles in vitro and in vivo [35,36,38]. Soluplus^®^ is a hydrophilic graft copolymer, which can easily form colloidal micelles with very good solubilization ability and stability, due to its low value of CMC (0.76 × 10^−3^%, 0.0003 mM) [53,54]. Solutol^®^ HS15 has a CMC of 0.005–0.02% (0.06 mM in distilled water) [55]. Thanks to its unique solubilization characteristics and safety profiles, this excipient has been used in parenteral, oral, ophthalmic, macromolecule, and protein formulations. The CMC of each copolymer and the molar fraction of the polymers in SSM were considered to evaluate the self-aggregation capacity of mixed micelles by calculating the theoretical CMC [40,56]. The CMC value resulted 1.82 × 10^−3^ mM. The low CMC value ensures the TQ-SSM high stability in the blood stream upon extreme dilution.

The cloud point is the temperature at which a non-ionic surfactant solution becomes cloudy due to the two-step separation when the system is heated. The phenomenon is explained by the dehydration of the surfactant with increasing temperature and by a decrease in the solubility of the surfactant [57]. The cloud point helps to select the storage conditions and to predict the stability of the formulation after administration. In this work the cloud point of TQ-SSM was found to be 30.7 ± 0.4 °C (mean ± SD, n = 3) and the cloud point of empty SSM is 29.5 ± 0.5 °C (Mean ± SD, n = 3). Despite the not very high value, the system maintained the same size and homogeneity as resulted by DLS analyses. As previously reported [40,56], the cloud point of a system is influenced by the inter-micellar interactions of the polymers, and the physicochemical characteristic of the mixed micelles may substantially differ from that of single micelles. Soluplus has a cloud point of 40.7 ± 0.8 °C (Mean ± SD, n = 3) [40], while Solutol^®^ HS15 of 70.0 ± 0.7 °C (Mean ± SD, n = 3) [58].

### 2.3. In Vitro Release

**Figure 3:**
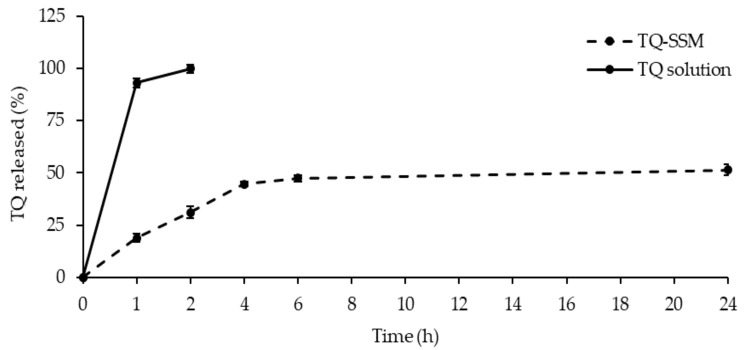
In vitro release profiles in PBS (pH 7.4) of TQ from aqueous saturated solution (TQ) and from micelles TQ-SSM.

The dialysis bag method was applied to study the release of TQ from micelles. PBS (pH 7.4) was used as a release medium, the test was carried out in the sink conditions. Maintaining a sink condition means keeping the drug concentration in a release medium low enough not to affect the concentration gradient for drug release. For this aim, at predetermined time intervals, 1 mL of release medium was withdrawn for HPLC analyses and replaced with the same volume of fresh release medium. As demonstrated by Figure 3, the immediate release of the drug was found for free TQ, while in the case of TQ-SSM the release of TQ takes place gradually until a maximum percentage of 51.50 ± 2.44% in 24 h. The almost linear and gradual trend of the release indicated that the SSM systems can release TQ for prolonged periods and in greater quantities compared to the saturated aqueous solution.

The results also revealed that TQ released from the polymeric micelle follows a slow and constant release rate compared to free TQ and after 6 h the percentage of drug release was in a plateau. However, SSM avoid rapid TQ release in the early hours unlike free TQ and this this slow release could prevent the degradation of TQ during the delivery and blood circulation.

### 2.4. Stability Storage

**Figure 4:**
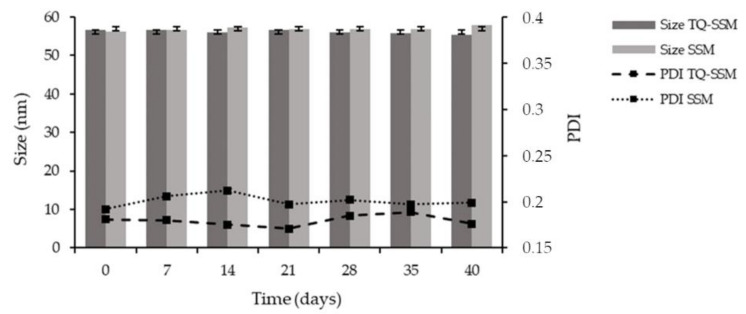
Particle size and polydispersity index (PDI) of TQ-SSM as dispersion after 40 days’ storage at 4 °C. Data displayed as mean ± SD (n = 3).

Stability studies on nanodelivery system represent an important issue for the applicability of the formulation in daily practice and thinking about an industrial production. The stability of TQ-SSM as dispersion was monitored during 40 days at 4 °C (Figure 4), because the stability at +25 °C was limited to 2 weeks, with a worsening of chemical stability. The physical parameters remained practically unchanged during the storage period. The size ranges from 56.71 ± 1.41 to 55.39 ± 1.13, PDI from 0.180 ± 0.003 to 0.176 ± 0.006 and zeta potential from −8.53 ± 0.27 to −7.81 ± 0.32. Also, regarding chemical stability, the EE% and DL% values remain the same even after 40 days, 90.23 ± 0.33 and 4.02 ± 0.14, respectively. No turbidity and layer separations were observed in the colloidal dispersion during the 40 days.

Furthermore, the stability study was performed on freeze-dried product (TQ-SSML) for 1 month at +25 °C. The solid product remains stable with sizes of 58.95 ± 2.31 and PDI of 0.205 ± 0.011. After the hydration, the formulation is easily re-dispersible, without leaving undissolved solid. Then, two possible ways to improve the stability of the micellar formulation is keeping the product in the fridge or eliminating water from the formulation.

### 2.5. Stability in the Presence of Human Serum Albumin (HSA)

**Figure 5:**
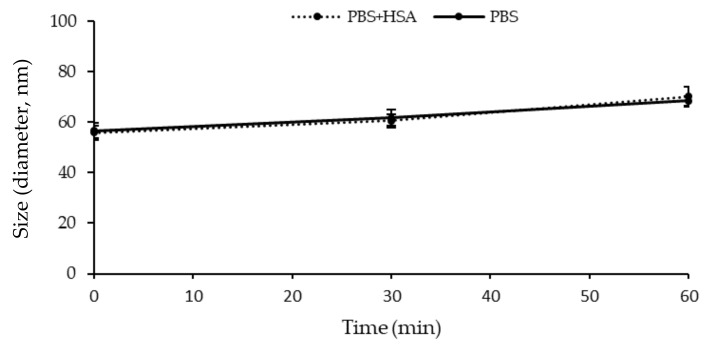
Stability of TQ-SSM in PBS and PBS + HSA for 1 h.

TQ-SSM showed a slight increase in terms of PDI and particle size when incubated with PBS in the presence of HSA compared to the condition of PBS only (Figure 5). This was probably due to the coexistence of albumin and micelles. However, it is evident that the formulation does not undergo drastic changes suggesting that the micelles are able to maintain their structure in physiological pH conditions and also in the presence of plasma proteins, as a result of the shielding effect of the PEG moiety. The same behavior was observed with empty micelles SSM: the average diameter ranges from 56.7 ± 1.3 nm to 71.95 ± 2.2 nm (Mean ± SD, n = 3) after 1 h when incubated with PBS and HSA.

### 2.6. TQ Effect on SH-SY5Y Cell Migration

**Figure 6:**
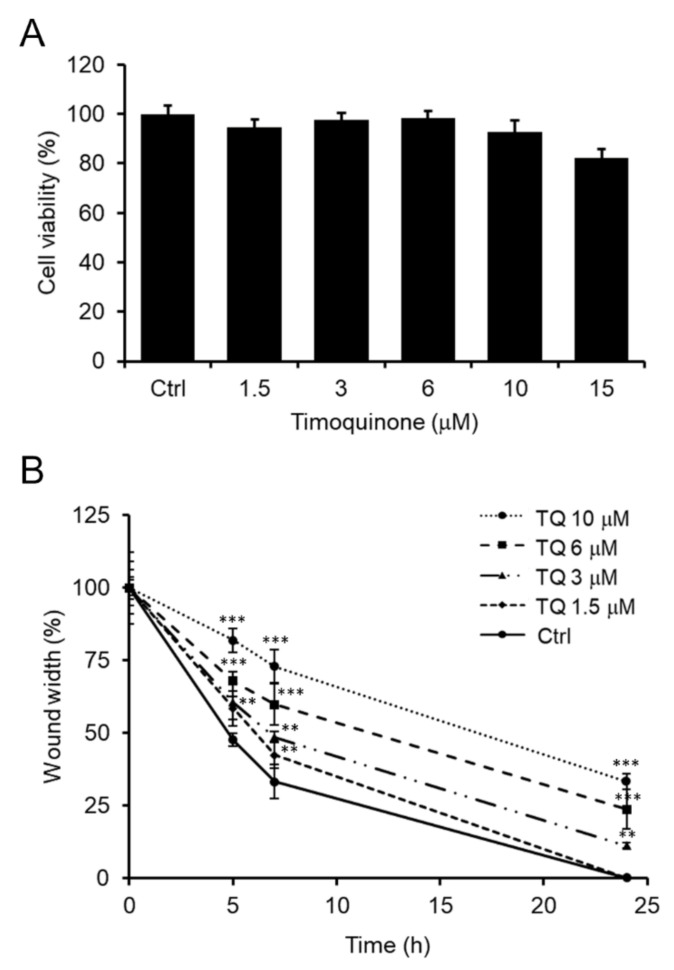
Effect of TQ on SH-SY5Y cell viability and migration. (**A**) MTT assay on cells treated with various TQ concentrations for 24 h under starvation condition. Untreated cells were used as control. Data are reported as percentage values compared to untreated control cells and expressed as the mean ± standard deviation of at least three independent experiments. (**B**) Time course analysis of the scratch closure of SH-SY5Y cells treated following the same experimental condition above described. Wound width values were measured considering the horizontal distance between the initial scratch and the scratch after migration at different time points and were reported as a percentage ratio with respect to the 0 h time point. Data are expressed as the mean ± standard deviation of at least three independent experiments. Error bars represent standard deviation. ** *p*-value < 0.01, *** *p*-value < 0.001 vs. untreated control cells.

The TQ effect on SH-SY5Y cell viability was determined by MTT assay. As illustrated in Figure 6A, TQ showed no signs of toxicity in terms of cell viability at concentrations from 1.5 to 10 μM while maintaining the cell viability level unchanged from that of untreated control cells. Only about 20 ± 4% reduction in cell viability was observed in cells treated with 15 μM TQ.

In the literature it is widely reported that TQ can suppress the migration of cancerous cells [12,13,14,15,16,17,18], including mouse neuroblastoma cancer cells [13]. For the first time this work explored the effect of TQ also on human SH-SY5Y neuroblastoma cell migration.

First, the effect of TQ on SH-SY5Y cell migration was observed in vitro using wound-healing assays. Cells were treated under starvation conditions with completely non-toxic TQ doses (1.5 to 10 μM) to rule out any possible cytotoxicity interference on cell migration events. The wound was performed on cell monolayers and cell migration was monitored at time intervals. Finally, the distance traveled by the SH-SY5Y cells in the scratch area has been quantitatively defined.

Specifically, TQ exhibited a dose-dependent ability to inhibit cell migration. As depicted in Figure 6B, as early as 5 h after the initial scratch, the wound width was approximately 80 ± 4% and 70 ± 3% of the initial width in cells treated with 10 and 6 μM of TQ, respectively, while untreated control cells migrated up to 50 ± 2% of wound width. Thus, TQ showed a slightly lower, but still effective inhibitory activity on cell migration at 3 and 1.5 μM concentrations (60 ± 6% and 60 ± 4% of wound width, respectively). The TQ inhibition activity remained reasonably high in subsequent time points. At 7 h from the initial scratch, cells treated with 10 and 6 μM of TQ migrated rather slowly, so the wound width was 73 ± 6% and 60 ± 7%, respectively (about 10% less width than 5 h), while untreated control cells migrated faster to a wound width of 30 ± 6% (20% less width than 5 h). Cells treated with 3 and 1.5 μM of TQ had an intermediate behavior, so at 7 h the wound width was respectively about 48 ± 2% and 42 ± 4% (approximately 15% less width than 5 h). After 24 h only cells treated with 1.5 μM TQ reached wound closure exactly as untreated control cells, while cells treated with the highest TQ doses (10, 6 and 3 μM) maintained a wound opening of 33 ± 3%, 24 ± 7%, 11 ± 1%, respectively. The doses of TQ, here considered effective, agree with the literature [59].

### 2.7. The Bio-Enhancement of TQ Activity Once Loaded into SSM Polymeric Micelles

Since mixed Soluplus^®^-Solutol^®^ HS15 micelles (SSM) are a standard and conventional nanoformulations widely used for application in biopharmaceutical studies [49,60] in this work the ability of SSM to improve the inhibitory role of TQ on human SH-SY5Y neuroblastoma cell migration was explored.

Hence, to investigate a potential bio-enhancement of TQ activity, TQ (5 mg/mL) was loaded into SSM polymeric micelles. Initially, to select the TQ-SSM doses at which the final biological effect could undoubtedly be attributed to TQ excluding any interference from SSM, the effect of empty SSM was examined on cell viability and migration. Then, SH-SY5Y cells were treated with empty SSM at 1:3000, 1:5000 and 1:10,000 dilutions, corresponding to the final loaded TQ concentrations of 10, 6 and 3 μM, i.e., the higher doses proven effective in inhibiting cell migration.

**Figure 7:**
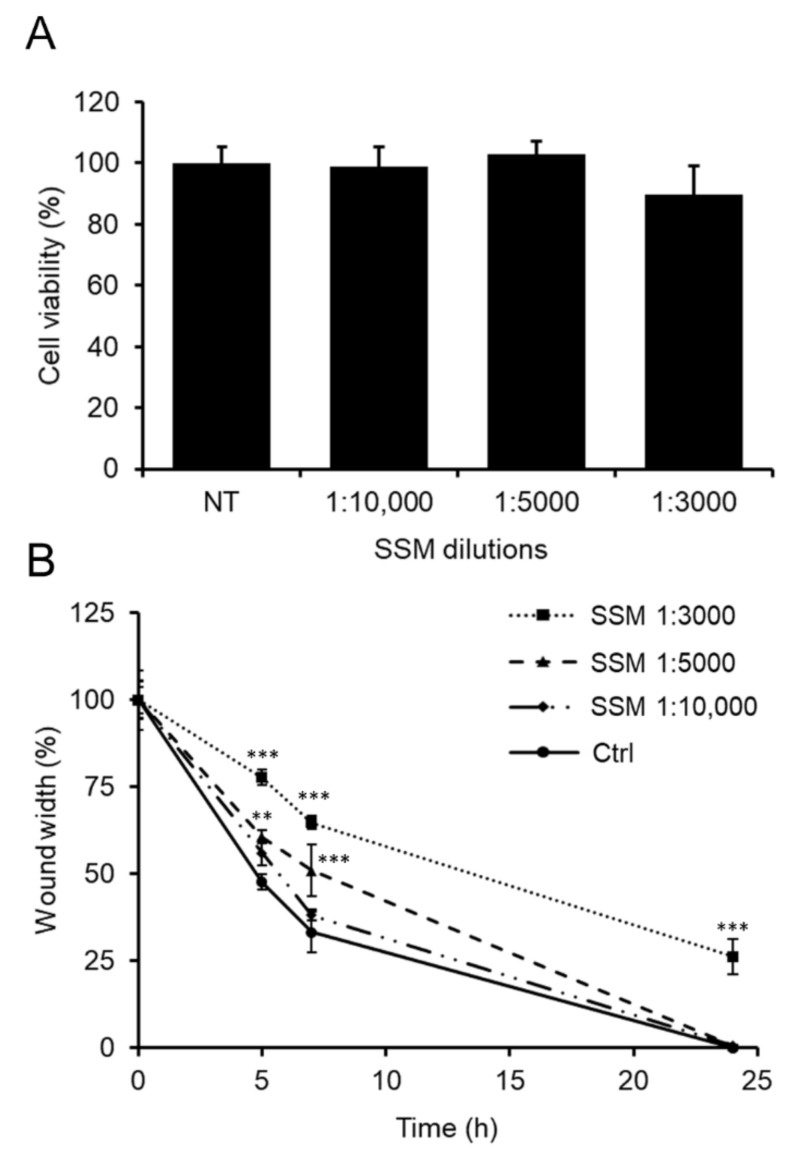
Effect of empty SSM on SH-SY5Y cell viability and migration. (**A**) MTT assay on cells treated with empty SSM at appropriate dilutions for 24 h under starvation condition. Untreated cells were used as control. Data are reported as percentage values compared to untreated control cells and expressed as the mean ± standard deviation of at least three independent experiments. (**B**) Time course analysis of the scratch closure of SH-SY5Y cells treated following the same experimental condition above described. Wound width values were reported as a percentage ratio with respect to the 0 h time point. Data are expressed as the mean ± standard deviation of at least three independent experiments. Error bars represent standard deviation. ** *p*-value < 0.01, *** *p*-value < 0.001 vs. untreated control cells.

Although empty SSM did not affect cell viability, which remained comparable to that of untreated control cells (Figure 7A), a noticeable impact of SSM 1:3000 on cell migration was nevertheless observed. As illustrated in Figure 7B, there was a slowdown in the migration of SSM 1:3000-treated cells as early as 5 h (77 ± 2% of wound width) which persisted even after 7 h (65 ± 2% of wound width) preventing the wound from closing after 24 h (26 ± 5% of wound width). Therefore, over time the wound width was constantly wider by about 30% compared to that of untreated control cells. At the higher dilutions 1:5000 and 1:10,000, the impact of empty SSM on cell migration was moderately reduced. In fact, in SSM 1:5000 or SSM 1:10,000 treated cells, the wound was only 5–10% wider over time (60 ± 2% and 56 ± 4% of wound width, respectively, at 5 h; 51 ± 8% and 38 ± 2% of wound width, respectively, at 7 h) compared to untreated control cells. However, this mild inhibiting effect was no longer evident at 24 h when wound closure was complete as in untreated control cells.

Knowing the action of SSM on cell viability and migration, we decided to test TQ-SSM at dilutions 1:5000 and 1:10,000, at which empty SSM showed no effects on cell viability and migration while the corresponding concentrations of unformulated 6 and 3 μM TQ showed efficacy in inhibiting cell migration.

**Figure 8:**
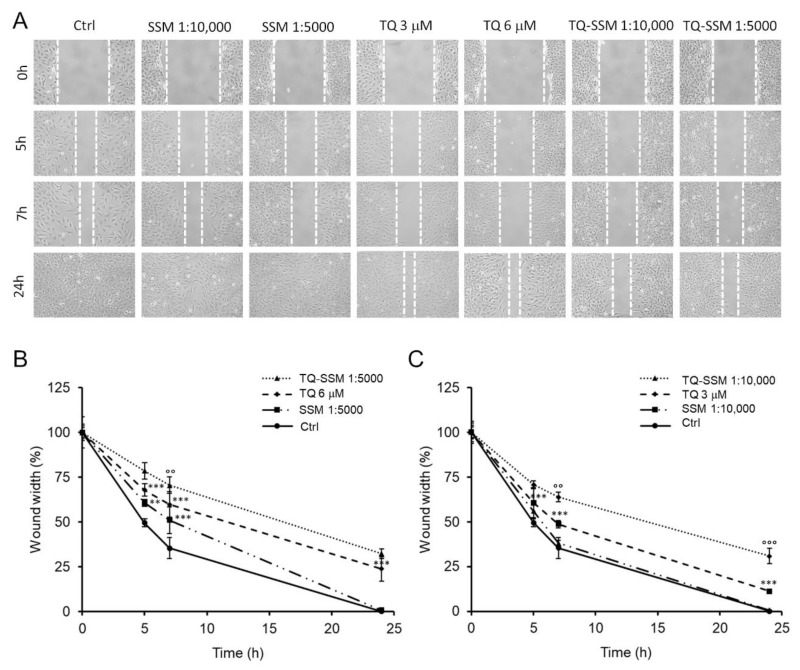
TQ-SSM effect on SH-SY5Y cell migration. (**A**) Representative image of SH-SY5Y cells, untreated or treated with empty SSM, TQ, or TQ-SSM for 24 h under starvation conditions. Scratch closure was monitored over time in cells. The dashed lines mark the edges of the wound area. (**B**) Time course analysis of the scratch closure of SH-SY5Y cells treated with TQ-SSM 1:5000, TQ 6 μM or SSM 1:5000. (**C**) Time course analysis of the scratch closure of SH-SY5Y cells treated with TQ-SSM 1:10,000, TQ 3 μM or SSM 1:10,000. Wound width values were reported as a percentage ratio with respect to the 0 h time point. Data are expressed as the mean ± standard deviation of at least three independent experiments. Error bars represent standard deviation. ** *p*-value < 0.01, *** *p*-value < 0.001 vs. untreated control cells; °° *p*-value < 0.01, °°° *p*-value < 0.001 vs. TQ-treated cells.

Figure 8A shows representative images of the wound-healing assay on cells treated with TQ-SSM. As illustrated in Figure 8B, cells treated with TQ-SSM 1:5000 migrated more slowly to a wound width of 79 ± 4% at 5 h, 70 ± 4% at 7 h and 32 ± 2% at 24 h compared to cells treated with unformulated 6 μM TQ. These data suggest that TQ-SSM 1:5000 allowed to improve the TQ inhibitory activity by approximately 10%. A very similar trend occurred for cells treated with TQ-SSM 1:10,000 (Figure 8C). At 5 h the wound width was 70 ± 2%, approximately 10% wider than that of cells treated with unformulated 3 μM TQ. Even at 7 h TQ-SSM 1:10,000 improved the inhibition of cell migration by about 15% (65 ± 3% of the wound width) compared to cells treated with unformulated 3 μM TQ and about 20% at 24 h (30 ± 4% of the wound width).

Overall, these data highlight that the loading of TQ into SSM polymeric micelles allows a bio-enhancement of TQ anti-migration activity, suggesting that TQ-SSM is a better candidate than unformulated TQ to inhibit human SH-SY5Y neuroblastoma cell migration. Probably, the carrier influences not only the solubility, but also the permeability of the TQ, providing an increase of its activity.

To date, cancer nanotechnology as a means of providing anticancer drugs is a promising strategy for cancer treatment. Our interesting results on the bio-enhancement of TQ activity once loaded into SSM suggest that the application of nanotechnology could represent a successful strategy for future clinical translation. Interestingly, the ability of nanotechnology to improve TQ bioactivity could be exploited as a strategic tool for the known therapeutic uses of TQ.

## 3. Materials and Methods

### 3.1. Chemicals and Reagents

BASF (Badische Anilin- und Soda Fabrik, Ludwigshafen, Germany) with the support of BASF Italia and BTC Chemical Distribution Unit (Cesano Maderno, Monza, and Brianza, Italy) kindly provided Soluplus^®^ and Solutol^®^ HS15. Distilled water was obtained from a Simplicity^®^ UV Water Purification System, Merck Millipore (Merck KGaA, Darmstadt, Germany). Thymoquinone, Phosphate buffered saline BioPerformance Certified pH 7.4 (PBS), Tween^®^ 80, lecithin (≥99%, TLC) lyophilized powder, cholesterol BioReagent (≥99), Methanol HPLC grade, Acetonitrile HPLC grade, Dimethyl sulfoxide (DMSO) HPLC grade, Formic acid analytical grade, 1,7-octadiene (98%), Dichloromethane (CH2Cl2) were purchased from Sigma–Aldrich (Milan, Italy). Sigma–Aldrich Merck (Milan, Italy) also supplied Dulbecco’s modified Eagle’s medium (DMEM), Ham’s F-12 nutrient mixture, fetal bovine serum (FBS), L-glutamine, penicillin and streptomycin, 1-(4,5-dimethylthiazol-2-yl)-3,5-diphenyl formazan (MTT) and other chemicals. Disposable plastics were obtained from Sarstedt (Nümbrecht, Germany).

### 3.2. Preparation of Thymoquinone-Loaded Mixed Micelles (TQ-SSM)

TQ-SSM were prepared by the thin film hydration method [40]. Briefly, Soluplus^®^ (400 mg), Solutol^®^ HS15 (400 mg) and TQ (25 mg) were dissolved in 10 mL CH_3_OH/CH_2_Cl_2_ mixture (1:4 *v*/*v*). Then, solvents were left to evaporate at 30–35 °C under vacuum for 20 min, until the formation of a film. Then, the film was hydrated with 5 mL of distilled water under sonication for 5 min to form a micellar dispersion. Blank mixed micelles SSM were prepared according to the aforementioned procedures without adding TQ.

### 3.3. Physical Characterization of TQ-SSM

The physical characterization was defined using Dynamic Light Scattering (DLS) by using Zsizer Nanoseries ZS90 (Malvern Molecules Instrument, Worcestershire, UK) at 25 °C for the determination of average diameter and the size distribution (polydispersity index, PDI) and Electrophoretic Light Scattering technique (ELS) employing the same instrument to assess the zeta potential. The results were expressed as the average of three measurements.

### 3.4. Drug Loading and Encapsulation Efficiency

The drug loading (DL%) and encapsulation efficiency (EE%) were determined by membrane filtration method [49]. TQ-SSM were filtered with a 0.20 µm filter membrane. The non-encapsulated TQ was retained on the membrane, while 20 µL of the filtrate was disrupted with 980 µL of MeOH.

The amount of TQ encapsulated and loaded into polymeric micelles was quantified by HPLC: the mobile phase consisted of (A) formic acid/water pH 3.2 and (B) acetonitrile. The flow rate was set at 0.8 mL/min. The gradient profile was: 0.10–25 min 10–90% B, 25–27 min 9% B, 27–30 min 10% B. For the calibration curve, different concentrations ranging from 0.002 µg/µL to 0.99 µg/µL were used. The linear correlation coefficient was >0.999. The DL% and EE% were calculated by equations (1) and (2), respectively:

(1) $$\mathrm{DL}\%=\frac{\mathrm{Weight}\;\mathrm{of}\;\mathrm{TQ}\;\mathrm{in}\;\mathrm{nanomicelles}}{\mathrm{Weight}\;\mathrm{of}\;\mathrm{fed}+\mathrm{Weight}\;\mathrm{of}\;\mathrm{the}\;\mathrm{excipients}}\times 100$$

(2) $$\mathrm{EE}\%=\frac{\mathrm{Weight}\;\mathrm{of}\;\mathrm{TQ}\;\mathrm{in}\;\mathrm{nanomicelles}}{\mathrm{Weight}\;\mathrm{of}\;\mathrm{TQ}\;\mathrm{fed}}\times 100$$

### 3.5. Theoretical Critical Micellar Concentration (CMC_theor_)

The theoretical CMC (CMC_theor_) value for SSM was calculated using the following equation (3) [56,61].

(3) $$\frac{1}{{\mathrm{CMC}}_{\mathrm{theor}}}=\frac{X_{\mathrm{Soluplus}}}{{\mathrm{CMC}}_{\mathrm{Soluplus}}}+\frac{X_{\mathrm{Solutol}}}{{\mathrm{CMC}}_{\mathrm{Solutol}}}$$

X_Soluplus_ and X_Solutol_ are the molar fractions of Soluplus^®^ and Solutol^®^, and CMC_Soluplus_ and CMC_Solutol_ are the CMC values of Soluplus^®^ and Solutol^®^, respectively. X_Soluplus_ and X_Solutol_ were calculated by the ratio between the moles of the constituent and the total moles of the constituents of the mixture.

### 3.6. Cloud Point

For the determination of the cloud point, the glass tubes containing 4 mL of TQ-SSM or SSM were immersed in a water bath at room temperature. Then, the temperature increased and the change of the sample from clear to turbid was observed. After that, the micellar formulations were cooled down and the measurements were performed in triplicate [62,63].

### 3.7. Lyophilization

TQ-SSM were frozen with liquid nitrogen and then placed into the freeze-drier Leybold Heraeus Lyovac GT2 equipped with a display for vacuum and temperature (Leybold GmbH, Cologne, Germany). The lyophilization was performed at a reduced pressure of 1 atmosphere for 24 h. The lyophilized samples (TQ-SSML) were placed in a silica gel desiccator at room temperature until the analysis. The average particle hydrodynamic diameter, the homogeneity, the surface charge and the EE% were determined after reconstitution of the powder with the original volume of distilled water. To ensure the complete dispersion of the powder the samples were vortexed for a few minutes.

### 3.8. Stability Studies

#### 3.8.1. Storage Stability Studies

The physical and chemical stability of TQ-SSM was investigate. The micellar dispersion is kept straight after their preparation without a further step and it was transferred into glass bottles sealed with plastic caps and stored at 4 °C over a period of 40 days while freeze-dried product (TQ-SSML) was stored at room temperature for 30 days.

The average size, PDI, zeta potential, EE%, DL% and any the clarity condition of the micelles systems were observed every week to evaluate the storage stability. Also, in the case of TQ-SSML the appearance of the powder and the redispersibility time were evaluated. The physical and chemical characterization was determined after reconstitution of the powder TQ-SSML with the distilled water.

#### 3.8.2. Stability in Blood Conditions

The average diameter and size distribution of TQ-SSM and SSM were evaluated after 1 h of incubation in PBS (pH 7.4) in the absence or in the presence of human serum albumin at a physiological concentration (Human Serum Albumin (HSA), 45 mg/mL). The samples were diluted into the media to obtain a HSA concentration of 0.5 mg/mL, then were incubated for 1 h at 37 °C under shaking (250 rpm). At scheduled time points (30 min, 1 h), aliquots of the samples were collected for DLS analyses [64,65]. The assays were performed in triplicate.

### 3.9. In Vitro Release Study

The in vitro release behavior of the TQ-SSM was studied using the dialysis bag method [40]. Two mL of TQ-SSM were added to a dialysis membrane (regenerated cellulose, Spectrum Laboratories Inc., Breda, The Netherlands, MWCO 3,5 kD) and immersed in 200 mL of the release media at 37 °C under magnetic stirring. PBS with 0.5% Tween^®^ 80 was selected as release media. The released TQ was under the sink conditions. Maintaining a sink condition means keeping the drug concentration in a release medium low enough not to affect the concentration gradient for drug release. For this aim, at predetermined time intervals, 1 mL of release medium was withdrawn for HPLC analyses and replaced with the same volume of fresh release medium. The release was monitored during 24 h. All experiments were performed in triplicate.

### 3.10. Cell Line and Treatment Conditions

SH-SY5Y human neuroblastoma cells were purchased from American Type Culture Collection (ATCC^®^, Manassas, VA, USA). Cells were grown in a 1:1 mixture of DMEM and Ham’s F12 supplemented with 2 mM L-glutamine, 100 μg/mL streptomycin, 100 U/mL penicillin and 10% FBS (complete medium), at 37 °C in a humidified atmosphere containing 5% CO_2_. At 90% confluence cells were appropriately propagated after trypsinization (trypsin 0.025%-EDTA 0.5 mM).

Wound-healing assays were performed on SH-SY5Y cells in serum-free medium (starvation medium) for 24 h in the presence of unformulated TQ at the final concentrations of 1.5 μM to 15 μM. The TQ-loaded polymeric micelles (TQ-SSM) were examined at appropriate dilutions so that the concentrations of TQ loaded in SSM were the same as those of unformulated TQ. Both cells untreated and treated with empty SSM were used as controls.

### 3.11. Cell Viability Assay

The viability of SH-SY5Y cells was assessed with the MTT assay, adapted to the cells used in this work. Cells were seeded in a 96-well plate (with a density of 5 × 10^3^/well) in the complete medium and allowed to grow overnight. Thereafter, a 24-h cell treatment in starvation medium with TQ at variable concentrations and TQ-SSM at appropriate dilutions was performed. Moreover, the effect of empty SSM on cell viability was investigated and untreated cells were used as a control. Once removing the medium, 100 μL/well of an MTT solution (0.5 mg/mL) was applied. After 1 h of incubation within the dark at 37 °C and washing in PBS, cells were lysed using 100 μL/well of a lysis buffer consisting of 20% (*w*/*v*) sodium dodecyl sulfate (SDS) in 50% (*v*/*v*) *N,N*-dimethylformamide. Finally, absorption values were recorded at 595 nm using a microplate reader (iMARK, Bio-Rad, Philadelphia, PA, USA). All data were reported as percentage ratios with respect to untreated control cells.

### 3.12. Wound-Healing Assay

The wound-healing assay was used to evaluated SH-SY5Y cell migration into the wounded area [39,66,67]. Cells were seeded in 6-well plate at the density of 5 x10^5^ cells/well in complete medium and were let to grow until a confluence monolayer was reached. Then, a longitudinal scratch was generated in the cell monolayer using a sterile 200 μL pipette tip. Cell debris was removed by washing in PBS and cells were treated with TQ at the different concentrations or TQ-SSM opportunely diluted in the culture medium. The empty SSM was checked for its effect on cell migration. Untreated cells were used as control. Cell-free area was observed with phase contrast microscope and images were taken at a time interval of 0, 5, 7 and 24 h after the scratch using a Nikon TS-100 microscope equipped with a digital acquisition system (Nikon Digital Sight DS Fi-1, Nikon, Minato-ku, Tokyo, Japan). Data were analyzed by quantitatively measuring the distance traveled horizontally by SH-SY5Y cells between the edges marked along each wound. The wound width data at each time were expressed in percentage terms with respect to the width of the wound at the initial time.

### 3.13. Statistical Analysis and Graphics Preparation

The experiments were repeated three times and results were expressed as a mean ± standard deviation. The statistical analysis of cell assay was performed with Tukey’s test. Furthermore, the graphs were drawn using LibreOffice Calc. Panels were assembled with LibreOffice Impress and adapted with Gimp 2.8.

## 4. Conclusions

In the current study, Soluplus^®^ and Solutol^®^ HS15 mixed micelles was used as an optimal system to enhance the ability of TQ to inhibit cancer cell migration. The solubility of TQ increased of about 10-fold and the micelles have good physical parameters and high encapsulation efficiency. Their stability as colloidal dispersion was demonstrated for a 40 days’ period. The lyophilization process was considered to produce a dosage form with increased stability. The process does not alter physical and chemical characteristics of the micelles. Due to the encapsulation into SSM, TQ efficiency increased and the controlled drug release was improved. SSM are particularly useful for targeting water-insoluble drug administration. Moreover, results from the in vitro wound-healing model showed that SSM were able to improve the ability of TQ to inhibit human SH-SY5 Y neuroblastoma cell migration. As for all pharmacological investigations, a pre-clinical in vitro study is foreseen before proceeding to an in vivo experimentation. Therefore, given the achieved results the efficacy of TQ loaded into these nanoformulations will be further investigated through in vivo animal models.

Overall, the ability to increase solubility and improve the stability and permeability of poor lipophilic drugs makes polymeric micelles, also related to the constituents of carrier, a promising delivery system for parenteral administration.

## Figures and Tables

**Table 1:** Physical and chemical properties of empty SSM and TQ-SSM. All values are expressed as means ± standard deviation.

	Particle Size (nm)	Polydispersity Index (PDI)	Zeta Potential (mV)	EE (%)	DL (%)
SSM	49.25 ± 1.92	0.183 ± 0.028	−5.46 ± 0.38	-	-
TQ-SSM	56.71 ± 1.41	0.177 ± 0.007	−8.53 ± 0.27	92.4 ± 1.6	4.68 ± 0.12
TQ-SSML	52.12 ± 1.18	0.183 ± 0.00	−7.89 ± 0.32	89.8 ± 0.2	4.11 ± 0.17
